# Complex magnetic fields represent an eco-sustainable technology to counteract the resistant *Candida albicans* growth without affecting the human gingival fibroblasts

**DOI:** 10.1038/s41598-023-49323-7

**Published:** 2023-12-12

**Authors:** Silvia Di Lodovico, Morena Petrini, Emira D’Amico, Paola Di Fermo, Firas Diban, Sara D’Arcangelo, Adriano Piattelli, Luigina Cellini, Giovanna Iezzi, Mara Di Giulio, Simonetta D’Ercole

**Affiliations:** 1 Department of Medical Oral and Biotechnological Sciences, University “G. d’Annunzio” Chieti-Pescara, Via dei Vestini, 31, 66100 Chieti, Italy; 2 Department of Pharmacy, University “G. d’Annunzio” Chieti-Pescara, Via dei Vestini, 31, 66100 Chieti, Italy; 3 School of Dentistry, Saint Camillus International, University of Health and Medical Sciences, Via di Sant’Alessandro 8, 00131 Rome, Italy; 4 Facultad de Medicina, UCAM Universidad Catolica San Antonio de Murcia, 30107 Murcia, Spain

**Keywords:** Microbiology, Health care, Physics

## Abstract

Novel technologies such as complex magnetic fields—CMFs represent an eco-sustainable proposal to counteract the infection associated to resistant microorganisms. The aim of this study was to evaluate the effect of two CMF programs (STRESS, ANTIBACTERIAL) against clinical antifungal resistant *C. albicans* also evaluating their uneffectiveness on gingival fibroblasts (hGFs). The STRESS program was more efficacious on *C. albicans* biofilm with up to 64.37% ± 10.80 of biomass and up to 99.19% ± 0.06 CFU/ml reductions in respect to the control also inducing an alteration of lipidic structure of the membrane. The MTT assay showed no CMFs negative effects on the viability of hGFs with a major ROS production with the ANTIBACTERIAL program at 3 and 24 h. For the wound healing assay, STRESS program showed the best effect in terms of the rate migration at 24 h, showing statistical significance of p < 0.0001. The toluidine-blue staining observations showed the typical morphology of cells and the presence of elongated and spindle-shaped with cytoplasmic extensions and lamellipodia was observed by SEM. The ANTIBACTERIAL program statistically increased the production of collagen with respect to control and STRESS program (p < 0.0001). CMFs showed a relevant anti-virulence action against *C. albicans*, no cytotoxicity effects and a high hGFs migration rate. The results of this study suggest that CMFs could represent a novel eco-sustainable strategy to counteract the resistant yeast biofilm infections.

## Introduction

Nowadays, the antimicrobial resistance (AMR) and the development of effective regenerative procedures for the restoration of damaged tissues as a result of diseases or trauma, represent the main challenges in medicine. The worrying antimicrobial resistance/tolerance phenomenon reduced the traditional strategies effectiveness, causing around 1.27 million deaths in 2019^1^. The AMR is caused by natural mutations of bacteria and fungi, but also to the inappropriate use of antimicrobial substances in all areas of production, from medicine to crops, intensive farming with the consequent of effectiveness loss of common antibiotics and antifungal substances^1^. Regarding the most recent resistance to yeasts, the prolonged use of antifungal drugs affected the eradication rate, making the drug less effective in the management of invasive fungal infections^2^. These infections are associated with high morbidity and mortality and can be related with healthcare-associated transmission. In particular, the prolonged used of antifungal therapy for the oral candidiasis has led to *C. albicans* adaptation to the drugs developing resistance/tolerance and representing a global challenge for oral treatment^3^. In oral cavity, *C. albicans*, an opportunistic pathogen, can be found on the palate, vestibular buccal mucosa, tongue, sublingual tissue, saliva, teeth, gingiva and implants and it is responsible for different oral diseases such as oral candidiasis, hard and soft tissue infections. Most of these diseases are associated with *C. albicans* mono and poly-microbial biofilm also characterized by complex interaction with bacteria and host. The biofilm reduces the antifungal substance efficacy to the matrix, induces the phenotypic changes in the cells, and activation of resistance genes^4,5^. *C. albicans* rarely exists as monospecies biofilm and its hyphae facilitate the interconnection with other oral microrganisms such as *Streptococcus mutans* and *Enterococcus faecalis*^6,7^ forming an interkingdom consortium. O’Donnel et al. believe that poly-microbial interaction can synergize the pathogenic potential of one or the other microorganisms and oral cavity plays an important role as incubator of this complex “microbial soup”^5^. *C. albicans* plays an important role in the development in oral candidiasis that could present in different forms according to different conditions^6^. Pseudomembranous candidiasis causes the exfoliation of the mucosa, and it is most frequently associated with steroid therapy and immunocompromising disease. Acute erythematous candidiasis is associated to antibiotic therapy and consequently with microbial reduction, induced red, painful lesions of mucosa. Chronic erythematous candidiasis is mainly associated to dental prosthesis and low oral hygiene and chronic hyperplastic candidiasis (CHC) is usually associated with smoke and it is the most dangerous form because can degenerate in a neoplastic lesion. Cieplik et al. reported that chlorhexidine rinses could induce formation of “persisters” in *C. albicans* biofilms, correlated with a higher risk of developing candidiasis, particularly in immunocompromised patients^8^*,* in which a percentage of 55% is reached. The causes are related to many factors such as long-term hospitalization, acquired immunodeficiency (HIV infection), treatment-induced immunodeficiency in patients receiving organ transplants, and conditions like malignancies, chemotherapy, and radiotherapy that compromise the cell mediated immunity and lead to mucosal disruption favouring the fungal infections^9–11^. In order to assure the healing of the tissue, in all these types of lesions, it is essential to balance the *C. albicans*, proliferation in the oral cavity without affecting healthy cells. Thus, it is crucial to modulate *C. albicans* presence in oral cavity, its biofilm formation and hyphae production, in order to achieve the successful treatment as well as implementing advanced eco-sustainable strategies, effective in eliminating planktonic microorganisms and their biofilms within this environment.

A global action plan was approved at the 68th World Health Assembly in May 2015 in order to address the growing problem of antimicrobial resistance/tolerance^12^. The goal is to develop new efficient strategies with low environmental impact, including the contribution provided by new technologies, such as complex magnetic fields (CMFs). These are generated by electrical energy of different voltage and therapeutic treatments can be produced, different in frequency, intensity, duration of exposure, pulses and waveform. The CMFs are able to affect different biological process including bone regeneration after trauma and Orthopedic surgery, musculoskeletal chronic pain and to enhance angiogenesis^13–15^. Zanotti et al. demonstrated that CMFs induced a ROS reduction, increased the macrophage M2 anti-inflammatory phenotype, reduced the inflammatory cytokines such as IL-1 and IL-6, increased the anti-inflammatory ones like IL-10 and IL-12 and improved the wound healing without a risk for the human health^16^. In fact, the exposition to the EMFs in the range of the interest don’t cause a negative effect on the patient and on the operator or other people according to the International Commission on Non-Ionizing Radiation Protection guidelines. The exposure to extremely low-frequency magnetic fields inhibits the proliferation of pathogenic microorganisms, such as *Salmonella*, *E. coli* or *C. albicans*^17,18^. The alternating magnetic field of 50 Hz affected the *C. albicans* viability, representing a new strategy for *Candida* infections^19^. D’Ercole et al., in previous two studies, demonstrated significant antimicrobial/anti-virulence actions of different CMF programs against *C. albicans* ATCC 10231, suggesting to better investigate the effect against clinical resistant *C. albicans* strain^20,21^. Based on these considerations, the aim of this study was twofold: (1) to evaluate the anti-biofilm effect of two different CMFs programs (STRESS and ANTIBACTERIAL) against a clinical resistant *C. albicans* strain, and their effect on the fluidity and permeability membrane changes; (2) to evaluate the effects of two different CMFs programs on gingival fibroblasts (hGFs) in order to verify if this technology exerts an effective antifungal activity against biofilm, without damaging gingival fibroblasts. The results of this study may suggest an eco-sustainable and safe strategy to manage the *C. albicans* biofilm infections in the oral cavity and could make contribution to the treatment of oral candidiasis. The results of this study could make a significant contribution to the treatment of oral candidiasis.

## Results

### CMFs anti-*C. albicans* S5 biofilm assays

**Figure 1:**
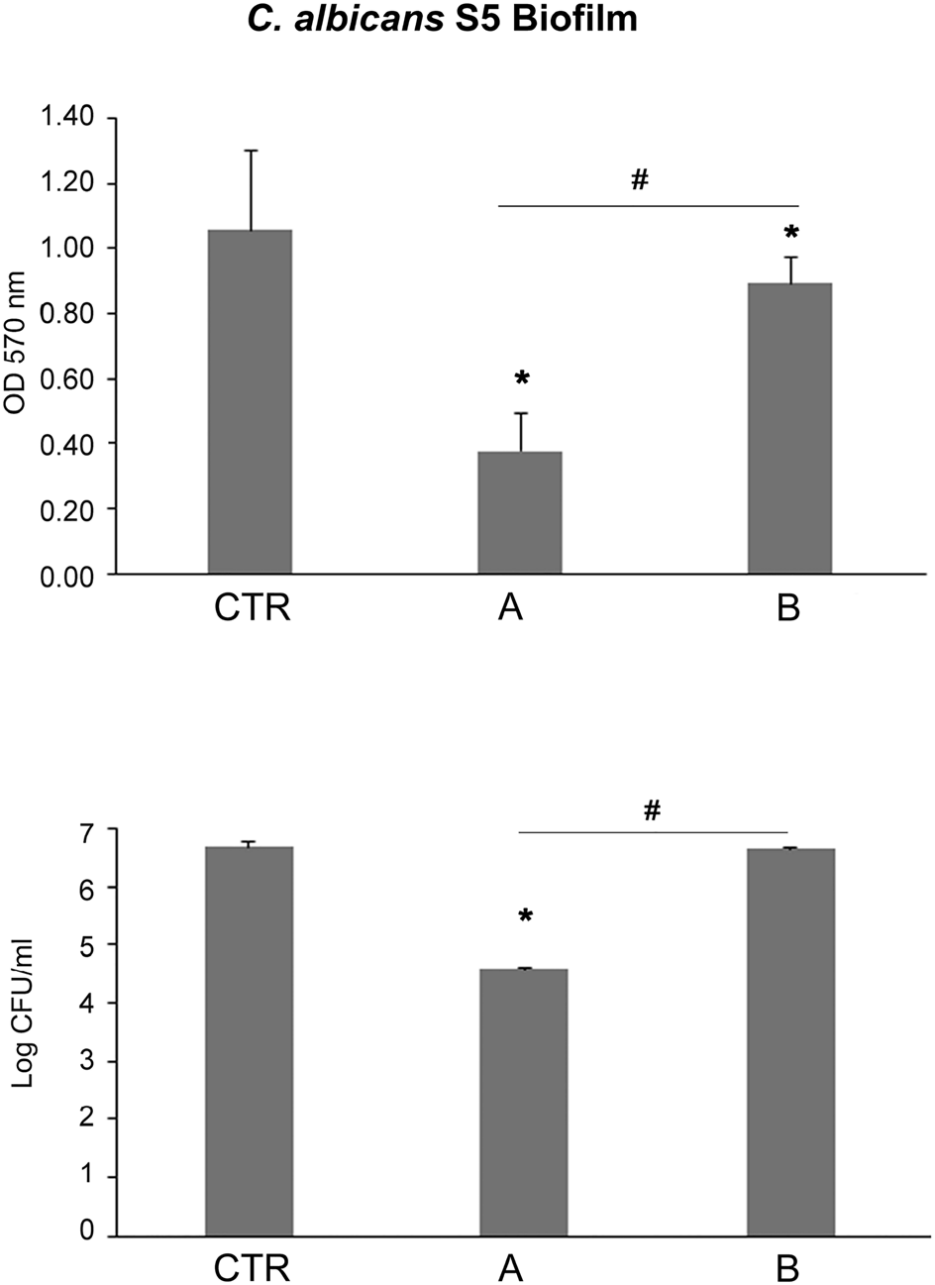
The CMFs effect on *C. albicans* S5 biofilm. up: *C. albicans* S5 biofilm biomass (OD 570 nm) production with and without (CTR) CMFs STRESS program (**A**) and ANTIBACTERIAL program (**B**). *Statistically (p < 0.05) significant in respect to the control; ^#^differences statistically (p < 0.05) significant. down: *C. albicans* S5 LogCFU/ml biofilm treated with and without (CTR) CMFs STRESS program (**A**) and ANTIBACTERIAL program (**B**). *Statistically (p < 0.05) significant in respect to the control; ^#^differences statistically (p < 0.05) significant.

The CMFs anti-biofilm effect was evaluated in terms of *C. albicans* S5 biofilm biomass production, colony forming units (CFUs)/ml, CLSM and SEM observation. CMFs showed a significant anti-biofilm activity against *C. albicans*. As shown in Fig. 1 up, CMFs STRESS and ANTIBACTERIAL programs significantly (p < 0.05) reduced the biomass production in respect to the control of 64.37% ± 10.80 and 15.63% ± 7.40, respectively. The best anti-biofilm effect was obtained with the STRESS program with a statistical (p < 0.05) significance in respect to the ANTIBACTERIAL program. In Fig. 1 down, reported was the *C. albicans* log CFU/ml of biofilm after treatments with CMFs for 22 min. In respect to the control, a significant CFU/ml reduction (p < 0.05) with both two tested programs were recorded. In particular, the best effect was obtained with the STRESS program reaching 99.19% ± 0.06 CFU/ml reduction in respect to the control (p < 0.05).

**Figure 2:**
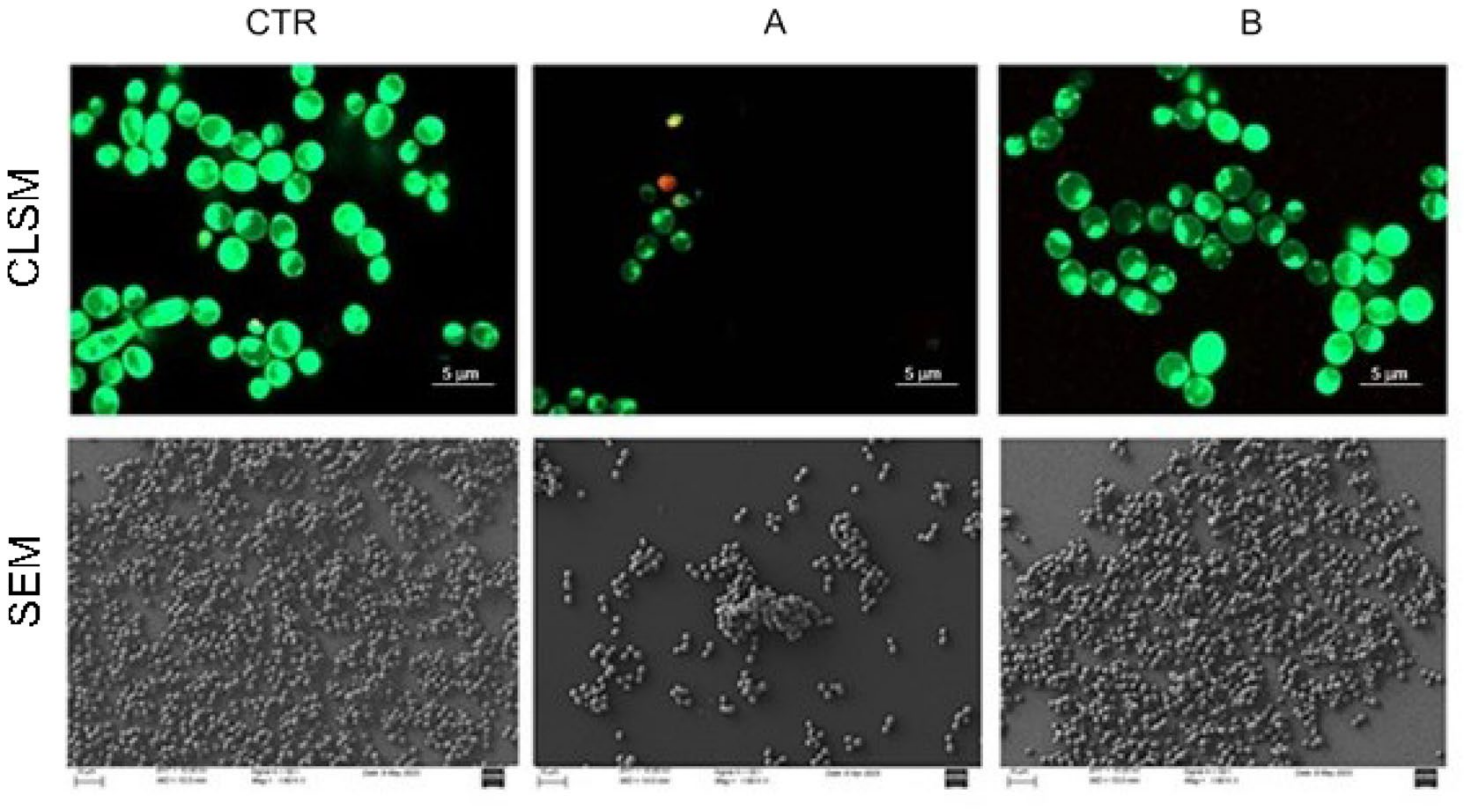
First line. Representative CLSM images of *C. albicans* S5 biofilm with and without (CTR) CMFs STRESS program (**A**) and ANTIBACTERIAL program (**B**). CLSM magnification ×63. Second line. Representative SEM images of *C. albicans* S5 biofilm with and without (CTR) CMFs STRESS program (**A**) and ANTIBACTERIAL program (**B**). SEM magnification ×1600 (scale bar 10 μm).

The CLSM images (Fig. 2 up) showed a not significant decrease of live fungal cells with the STRESS program. Results were significant in respect to controls and ANTIBACTERIAL program. On the contrary, a slight decrease in green living cells with the absence of red dead yeasts were found in the ANTIBACTERIAL program compared to the control. SEM images (Fig. 2 down) showed that CMFs reduced the *C. albicans* biofilm with a remarkable effect with STRESS program. Samples exposed to STRESS program were characterized by a significant reduction of *C. albicans* load. No morphological *C. albicans* changes were shown in both tested samples in respect to the control.

### Fluidity changes *C. albicans* S5 membrane

**Figure 3:**
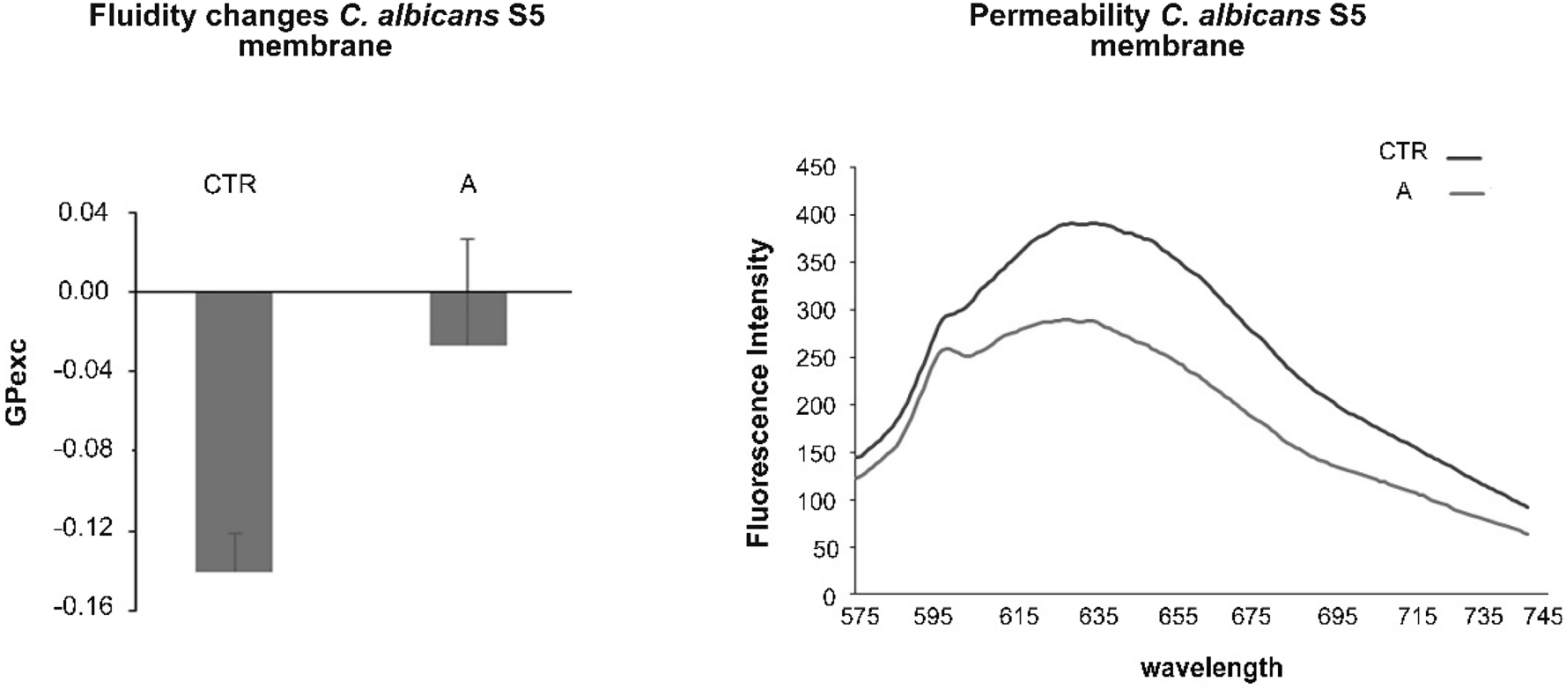
Fluidity *C. albicans* S5 membrane (left): *C. albicans* S5 GPexc with and without (CTR) CMFs STRESS program (**A**). Permeability *C. albicans* S5 membrane (right): *C. albicans* S5 permeability membrane with and without (CTR) CMFs STRESS program (**A**).

To evaluate the potential changes of *C. albicans* S5 fluidity membrane induced by CMFs STRESS program (A), after treatment, the Laurdan emission spectra and GPexc were evaluated. The experiments were performed by using CMFs STRESS program (A) that was the best one in the antibiofilm assay. As shown in Fig. 3 (left), the CMFs significantly (p < 0.05) reduced the fluidity of *C. albicans* membrane. The untreated *C. albicans* GPexc indicated a liquid-crystalline phases using an excitation wavelength of 340 nm and emission wavelengths of 440 and 490 nm. Higher Laurdan GPexc values in respect to the control correspond to lower membrane fluidity. The CMFs induced a reduction of membrane fluidity indicating a stress in the geometrical packing of the phospholipid membrane, a process governed by polar head group composition and fatty acyl chain conformation.

### Permeability ***C. albicans*** S5 membrane

The evaluation of *C. albicans* S5 permeability membrane changes was analysed by determination of fluorescence intensity of PI. The experiments were performed by using CMFs STRESS program (A) that was the best one in the antibiofilm assay. The Fig. 3 (right) reports the *C. albicans* permeability results obtained with the STRESS program. The decrease in fluorescence in treated samples indicates that PI did not entry into the *C. albicans* cells through ruptured bacterial membrane. The CMFs did not affect the permeability membrane change.

### Effects of CMFs on hGF density, morphology, viability and ROS production (Fig. 4)

**Figure 4:**
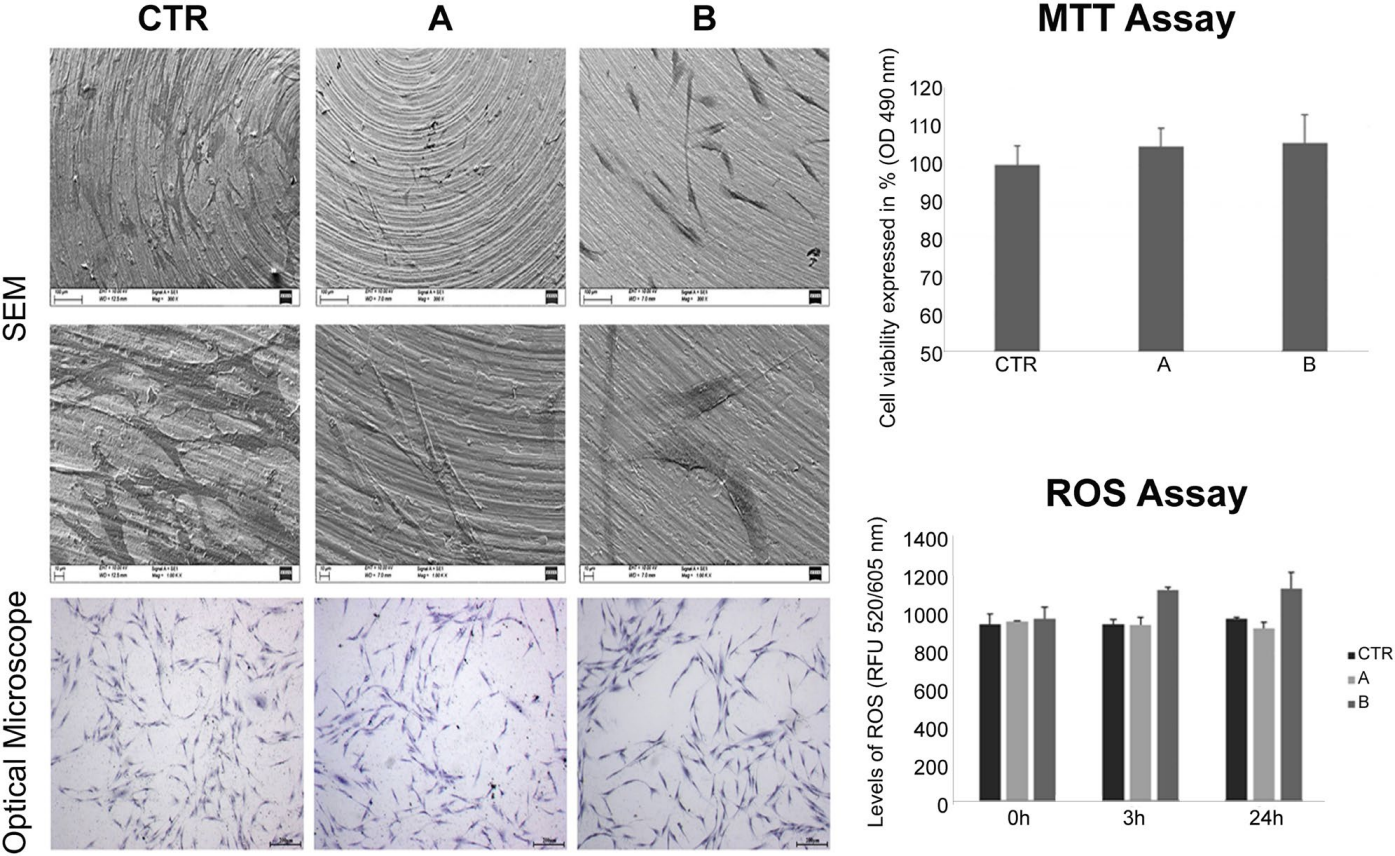
Effects of CMFs on hGFs density, morphology, viability and ROS production. hGFs morphology after CMFs treatment evaluated by SEM after 24 h. Magnifications first line: ×300 (scale bar 100 μm), and second line: ×1000 (scale bar 10 μm). Third line shows hGFs observed after toluidine blue staining at stereomicroscope. Magnification ×25, scale bar 200 μm. Upper histogram shows the effects of CMFs treatment (24 h) on hGFs viability. Values represent mean ± SD expressed in percentages compared to control (CTR). Lower histogram shows the levels of ROS quantified after the exposition to CMFs at T0, after 3 and 24 h from the treatment. Data are reported as mean ± SD of three independent experiments and expressed in the relative fluorescent unit (RFU), measured at λex/em 520/605 nm. *CTR* controls, (A) STRESS program; (B) ANTIBACTERIAL program. (**p < 0.001 vs CTR; ***p < 0.0001 vs CTR; ^##^p < 0.001 B vs A; ^###^p < 0.0001 B vs A).

SEM and blue toluidine staining were used to evaluate the hGFs density and morphology after the exposition to CMFs. The SEM observations showed that the hGFs colonized the surface at 24 h with the same density in group A and B. In treated (A,B) and control (CTR) groups the presence of elongated and spindle-shaped with cytoplasmic extensions and lamellipodia was observed mostly at 1000×. Regarding the toluidine blue staining, cell density was similar in test and control groups. In all conditions, hGFs exhibited the normal morphology of fibroblasts, characterized by a spindle shape. In addition, to evaluate the viability of gingival fibroblasts MTT assay was performed. The MTT assay (Fig. 4) showed that although cells exposed to CMFs were characterized by a slight increase in cell proliferation after 24 h, no significant differences with control were found. A and B groups showed a similar growth rate. The possible production of ROS was evaluated using a Cellular Reactive Oxygen Species Detection Assay Kit. ROS assay showed that program B induced a significant increase of ROS, in respect to the unexposed controls (CTR) and to cells exposed to the CMF program A after 3 h and 24 h from treatment. The treatment A showed similar values from 0 h at 3 h and 24 h and the levels were comparable to CTR. The levels of CTR were constant in the time.

### Effects of CMFs on wound healing

**Figure 5:**
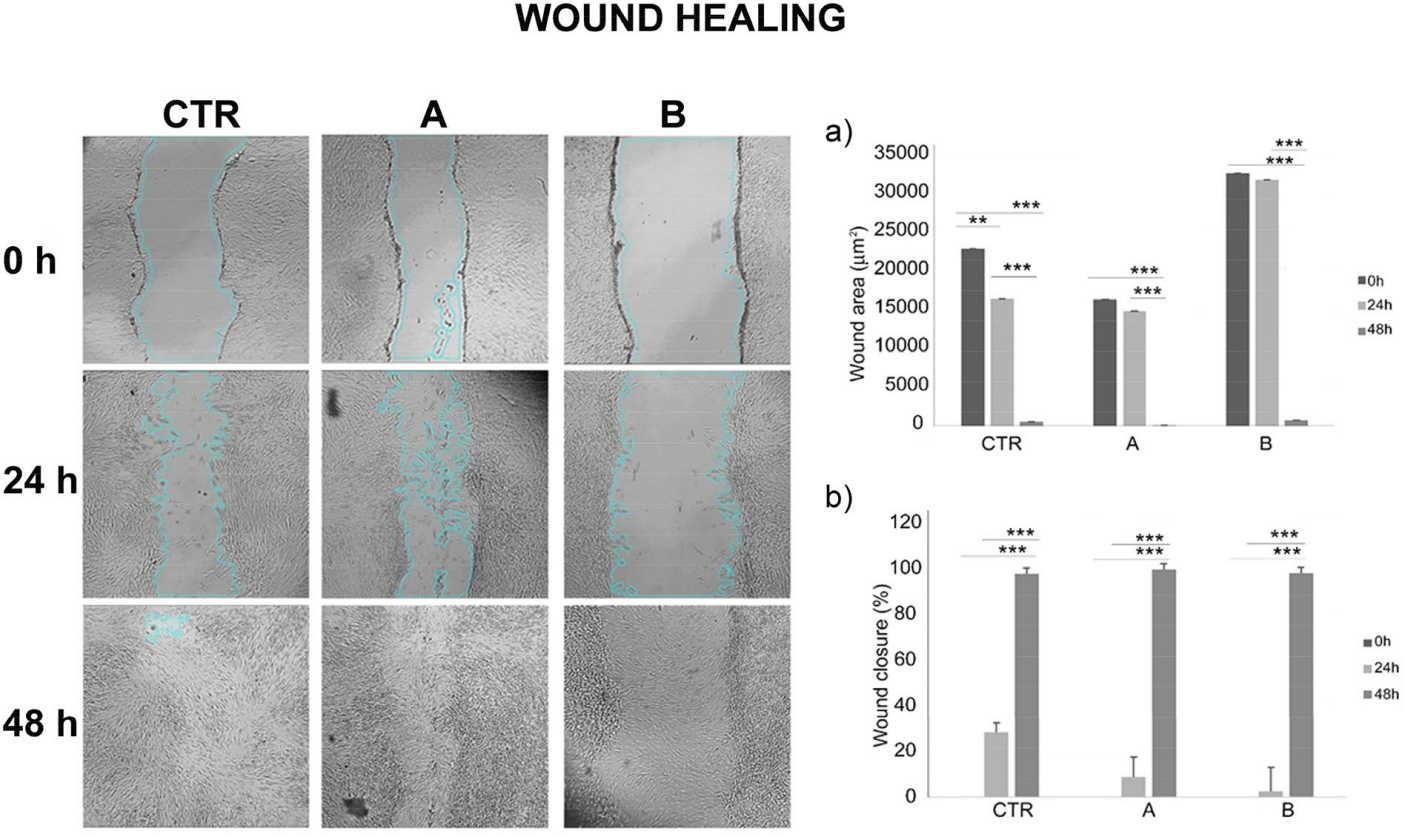
Effects of CMFs on the wound healing. Wound healing assay, in sky blue is shown the wound area. The images of the scratch were taken at 0, 24 and 48 h. Magnification ×4. The wound healing measurements comprehended the wound area expressed in μm^2^ (upper histogram), and the percentage of wound closure (lower histrogram). *CTR* controls, *A* STRESS program, *B* ANTIBACTERIAL program.

Wound healing assay was applied to test the ability of CMFs to induce the wound closure in hGFs. The images of the optical microscope of the wound healing showed that at 24 h both unexposed controls (CTR) and cells exposed to CMF STRESS program (treatment A) started the formation of bridges between each side of the wound, as a first step of closure. On the contrary, the ANTIBACTERIAL group (treatment B) showed no sign of healing after 24 h. At 48 h all groups showed a complete wound closure (Fig. 5). The measurements performed on the wound healing images (Fig. 5 upper and lower histograms) by Image J confirmed that after 24 h a decrease of the wound area values were observed. After 48 h, the wound area statistically decreased (Fig. 5 upper histogram). Given the difference width of the scratch, the wound closure, expressed in percentage, was calculated.The trend of wound aerea was the opposite in respect to the percentage of wound closure. It augmented in statistically significant manner after 24 and 48 h in both treatments (Fig. 5 lower histogram).

### Effects of CMFs on collagen production

**Figure 6:**
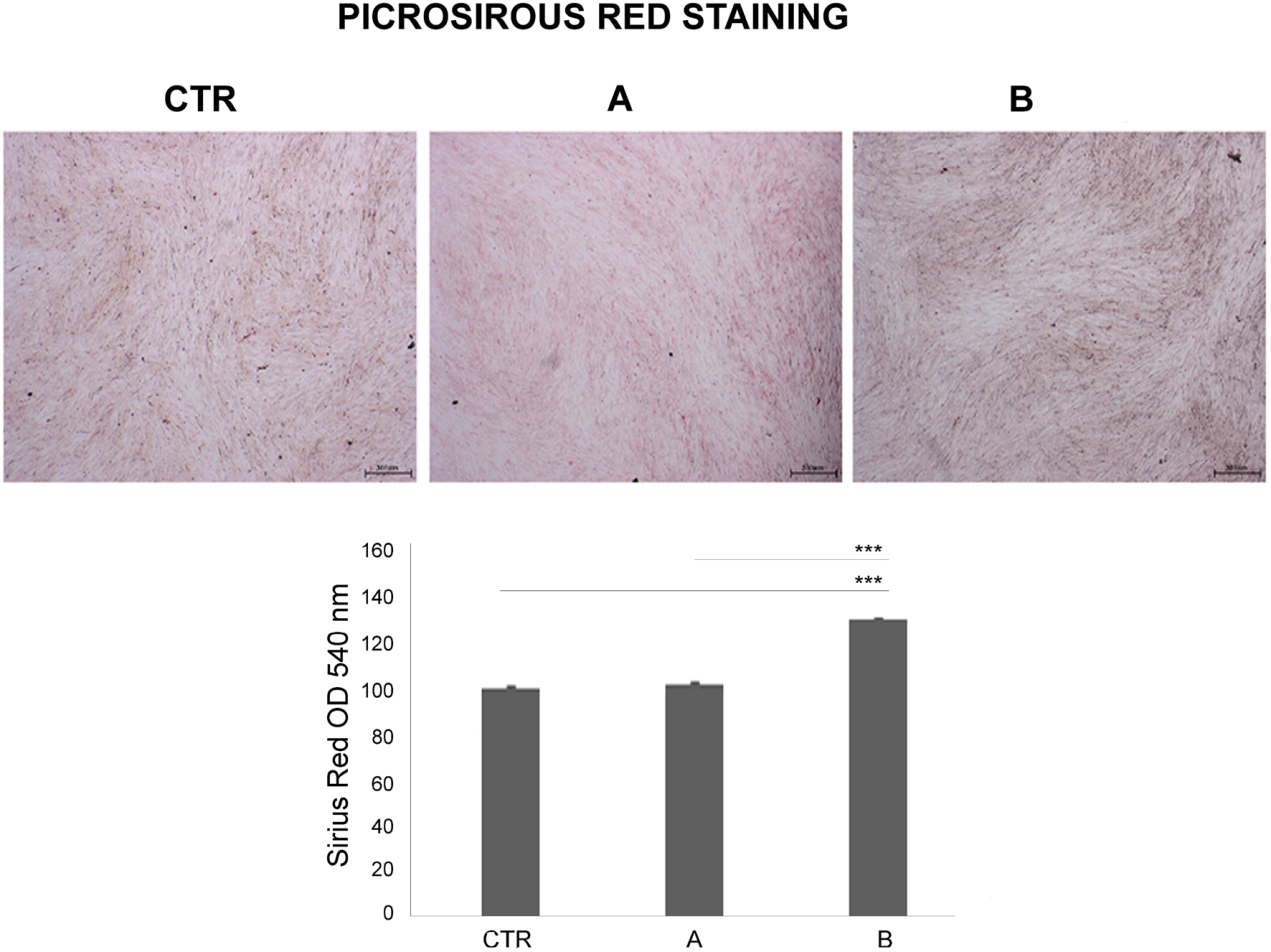
Effects of CMFs on collagen production. Picrosirius red staining observation (**A**) was quantified by spectrophotometric analysis at 540 nm (**B**). A: STRESS program; B: ANTIBACTERIAL program (***p < 0.0001 compared to CTR; ### p < 0.0001 B vs A). Magnification: ×25; scale bar 300 μm.

Picrosirius red staining was used to evaluate the collagen production (Fig. 6). The analysis was performed at 7 days and the observation at microscopy showed the presence of more intense red, tending to brown color, in the group B with respect to CTR and treatment A. These observations were confirmed by the spectrophotometric analysis. The histogram showed a significant higher production of collagen (p < 0.0001) in group B (ANTIBACTERIAL program) compared to treatment A (STRESS program) and CTR.

## Discussion

The worrying phenomenon of the antimicrobial resistance strongly suggests the search for new eco-sustainable approaches to counteract the infections associated with the resistant microorganisms. CMFs can be considered an eco-sustainable technology because their application do not need the use of adjunctive substances or drugs avoiding the disposal problem with no environmental impact being fully in line with the 2030 agenda for the sustainable development. Moreover, all the device components are easily washable and disinfectable and, therefore, reusable with different patients. D’Ercole et al. demonstrated that CMFs were able to induce antifungal, anti-virulence and antiadhesive effects on planktonic *Candida albicans* ATCC 10231, without affecting hGFs proliferation rates^20^. The aim of this study was to increase both the knowledge about the anti-virulence effects of CMFs against resistant *C. albicans* and their biological response to hGFs. CMFs reduced the resistant *C. albicans* biomass with a remarkable effect displayed particularly by the STRESS program. The capability of electromagnetic fields (EMFs) to affect the biofilm production, cell growth and viability depends on their physical parameters (frequency, magnetic flux density), the time of the exposure, and/or the type of bacterial cells used. Gerard et al. (2015) showed that the formation of biofilm under a very low-frequency EMF treatment was two times lower than unexposed biofilm^22^. However, other studies reported that low intensity and very low-frequency EMFs did not have significant impacts on biofilms in water distribution systems^22–24^. As shown by Xiao, the anti-biofilm effect of EMFs was related to their capability to interfere with the microbial quorum-sensing signaling (QS) regulating the biofilm production^25^. A remarkable anti-biofilm effect of CMFs STRESS program was displayed by the CLSM and SEM images, demonstrating the CMFs capability to disaggregate the *C. albicans* biofilm with a relevant killing action. As shown by fluidity measurements, STRESS program of CMFs induced a significant reduction of fluidity membrane that translated with a better organization of membrane’s phospholipids^26^. The CMFs affected the normal *C. albicans* fluidity increasing the rigidity of its membrane, as shown by the increase of GPexc values in respect to the controls. This effect suggests an unbalance of the microorganisms with a more susceptibility to the treatments. According to Di Lodovico et al., the increased rigidity identifies an effect on the cytoplasmic microbial membrane^26^. Alteration of membrane fluidity is associated with the formation of abnormal sphingolipid-ergosterol domains resulting in reduced microbial growth. This alteration could also be related to an effect on the chitin of the *Candida albicans* cell wall since it is responsible for the crystallization state of the cell. The STRESS program of CMFs did not affect *C. albicans* membrane permeability, as shown by the decrease in fluorescence in treated samples indicating that PI did not entry into the *C. albicans* cells. The results obtained in this study are not in contrast with previous studies on high-intensity pulsed electric fields (PEF), because CMFs apply very low intensity magnetic fields, comprised between 0.1 and 150–180 µT, which is almost comparable with the Earth's magnetic field that is comprised in the range of 20–70 µT^27^. On the contrary, high-intensity pulsed electric fields (PEF) that generate fields 5–6 orders of magnitude higher than the geomagnetic one, are able to induce an increase of membrane permeabilization and in particular with high-pulsed electromagnetic fields (PEMF) the effect follows a dose-dependent gradient^28–30^. PEMFs are able to induce magnetic fields a lower magnitude in respect to those of PEFs, although they are both able to induce electroporation^31^. Novickij et al. reported that the electric field variation was not the only parameter responsable for membrane permeabilization^27^ and analyzing the influence of different pulse shapes and parameters of PEMFs treatment (high dB/dt; B-magnetic flux density), concluded that PEMF induced an increased membrane permeabilization with a pulse in the sub-microsecond (450 ns) range because it was able to create a greater electric field^32^. The antifungal effects on *C. albicans*, but, at the same time, the lack of biological effects on hGFs, could be the results of the different elastic modulus of the eukariotic cells, in respect to bacteria and fungi. Indeed, as shown by Elbourne et al. the elastic modulus of fibroblasts is comprised in the range of 4–100 kPa, the osteoblasts 0.3–20.0 kPa^33^. On the contrary, *C. albicans* showed higher values of elastic modulus comprised between 150 and 190 kPa^34^. The application on magnetic fields on the cells causes the induction of vibrations, movement, spinning and rotation of the cells, with effects that can vary according to both the parameter of the protocol applied, but also by the biological characteristics of the cells. Indeed, the impact of magnetic fields on cellular membranes is dependent of their stiffness: the higher is the rigidity and the higher potential detrimental effects on the membranes^33^. The obtained results are in accordance with literature^1^. As shown by Rao et al. in 2022, cellular response is higly influenced by the parameters of the magnetic field, like the pulse width. Also small modifications of this parameter, from nanoseconds to milliseconds^35^, were associated with different patterns of intracellular compartments, and consequently, to different biological effects. The effects of CMFs on fibroblasts’s ROS production is dependent on the program applied, but also on the cellular status. Zanotti et al. have evaluated the ROS fluctuations after CMFs exposition on fibroblasts sampled from patients affected to diabetic foot. In non inflamed fibroblasts the ROS levels at 3, 5 and 7 days were constant, on the contrary the ROS significantly decreased in inflamed fibroblasts^36^. However, the program used by Zanotti et al. was “tissue regeneration”, that is different respect the programs applied in this study. The results showed a significant ROS increase at 3 and 24 h in fibroblasts exposed to program B, without recording any significant variations in respect to the controls. A chronic presence of high ROS levels is cytotoxic for cells, but as shown by the Picrosirius red staining, the higher collagen production shown by cell exposed to ANTIBACTERIAL program is a positive sign of healthy cells, so the ROS increase was probably only temporary, and did not affect cellular viability. Based on these results, STRESS program showed higher antifungal effects, whilst the ANTIBACTERIAL one an high ability to induce tissue regeneration, so for a possible clinical application of Candidiasis, in the first part of the treatment it would be better to apply first the ANTIBACTERIAL program, and then, for microbial removal, apply the STRESS program. The application of the new technologies such as CMFs could provide an innovative tool for the treatment of antibiotic-resistant microbial infections, and in particular for the treatment of biofilms associated to *C. albicans* infections, with a significant impact in terms of technological innovation and industrial applications. The use of CMFs satisfies the request of eco-friendly demand for the low environmental impact for their manufacturing, transport and disposal, respecting each production cycle.

Starting from the previous studies in which a remarkable antimicrobial action and an inhibition of the transition to the filamentous form were demonstrated against a reference strain of *C. albicans*,in this study, the anti-virulence effect of CMFs was demonstrated against a resistant strain of *C. albicans* in biofilm growth, suggesting their use as a valid strategy for the oral candidiasis associated to the yeast. The study limitation is related to the use of a single *C. albicans* strain and more resistant strains will be inserted in future studies to confirm the obtained data.

In conclusion, an important action of CMFs was shown both on *C. albicans* biofilm reduction and on wound healing. These significant effects were obtained without interfere with the hGFs proliferation. The biological effects of CMFs are also influenced by the program used for the treatment and by the cellular target that is exposed. The STRESS program is more efficacious on *C. albicans* biofilm with a remarkable action on the sessile growth with a relevant killing action. In addition, the STRESS program induces an alteration of lipidic structure of the membrane that results as highly organize and assembled. Both tested programs resulted safe, and the hGF proliferation was not affected, in respect to the controls. The ANTIBACTERIAL program was associated with an higher ROS production at 3 and 24 h. In addition, it is notable that there were a full wound closure at 48 h and an higher collagen production at 7 days. This may suggest that ROS production is a positive stimulus for cells. Based on these results, CMFs could be considered as an innovative, therapeutic approach based on the use of new technologies for the treatment of *C. albicans* biofilm infections. Finally, CMFs are defined as eco-friendly technology for their low environmental impact according to the 2030 agenda for the sustainable development.

## Materials and methods

### Experimental design

**Figure 7:**
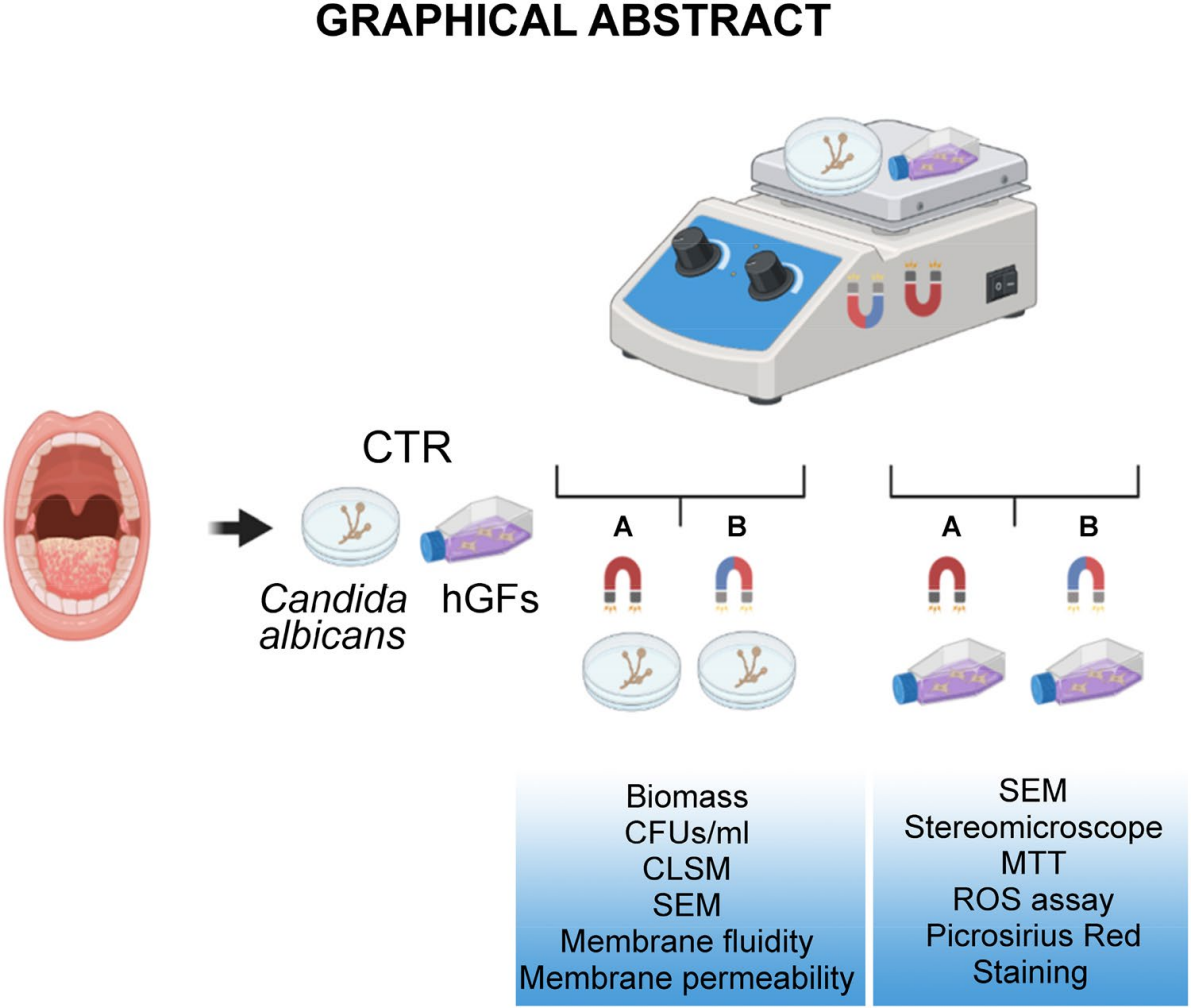
Experimental plan of the study. Clinical *Candida albicans* S5 strain and human gingival fibroblasts (hGFs) ATCC were treated with two different programs: A = STRESS and B = ANTIBACTERIAL. Figure created with BioRender.com.

*Candida albicans* and human gingival fibroblasts (hGFs) were exposed to CMFs according D’Ercole et al.^20^. The study was performed according the following experimental plan (Fig. 7). All methods were performed in accordance with the relevant guidelines and regulations.

### Microbial culture and biofilm preparation

Anonymised clinical *Candida albicans* S5 strain, isolated from oral cavity of a patient that gave his informed consent for the study, was used. The study (reference number: BONEISTO N. 22-10.07.2021, University G. d’Annunzio Chieti-Pescara, 10 July 2021) was approved by the Inter Institutional Ethic Committee of University “G. d’Annunzio” Chieti-Pescara, Chieti, Italy. The strain was characterized for the resistance profile against commonly antifungal drugs used in therapy^37^.For the experiments, *C. albicans* S5, grown on Sabouraud dextrose (SAB, Oxoid, Milan, Italy), was cultured in RPMI 1640 (Sigma-Aldrich, Milan, Italy) plus 2% glucose and standardized to OD_600_ = 0.15 (≈ 10^7^ CFU/ml). For the biofilm preparation, *Candida albicans* S5 was incubated on 96-well flat-bottomed microtiter plates at 37 °C for 24 h^38^. Subsequently, the planktonic cells were gently removed, and the wells were washed with sterile PBS and treated with CMFs as later described^20^.

### Human gingival fibroblasts (hGFs) cell culture

hGFs were obtained from ATCC (Manassas, VA, USA). The medium DMEM low glucose (Corning) supplemented with 10% fetal bovine serum (FBS) (SIAL), 1% penicillin, and streptomycin (Corning) were used to culture cells under condition of 37 °C and 5% CO_2_.

### Complex magnetic fields source (CMF)

The experiments were carried out in a 50 T magnetic field environment. *C. albicans* S5 biofilm and hGFs were exposed to multi-frequency magnetic fields with varying frequency, intensity, duration, and wave form. The device CMF machine, Next sx version (Medicina Fisica Integrata, M.F.I., Rome, Italy), creates pulsed electromagnetic fields between 0.1–250 µT^36^ and 1–250 Hz. The magnets are made by winding 650 turns of 0.35 mm-wide enamelled copper wire. The coil's external dimensions are 110 mm, internal dimensions are 12 mm, and the coil's thickness is 8 mm. Magnetic field lines are applied to the sample being treated at a 90° angle. In particular, two distinct programmes (A = STRESS and B = ANTIBACTERIAL) were used. These programmes were distinguished by different sequences of brief, single steps of complex magnetic fields (3–5 min each), with frequency ranging from 6 to 70 Hz, intensity from 6 to 95 µT, and complex waveforms with multiple harmonics.

### CMFs anti- ***C. albicans*** S5 biofilm assays

The CMFs effect on *C. albicans* S5 biofilm was evaluated in terms of: (1) biofilm biomass evaluation by crystal violet staining method; (2) CFU/ml count for the quantification of cultivable cells; (3) confocal laser scanning microscopy (CLSM) observation; (4) scanning electron microscope (SEM) analysis. *C. albicans* S5 biofilms were treated with CMFs at two differents programs: STRESS program (A) and ANTIBACTERIAL program (B)^20^. The treatment time was of 22 min. For the control, the *C. albicans* S5 biofilm did not received any treatment with CMFs. After treatment, for the biomass quantification, dry-biofilms were stained with 0.1% crystal violet and quantified according to Di Lodovico et al.^39^; for CFU/ml determination, the adhered *C. albicans* S5 cells were scraped off and resuspended in 200 μl of PBS, transferred to test tubes, vortexed for 2 min, diluted and spread on SAB and incubated for 24–48 h at 37 °C. Microscopic observations with Live/Dead staining prior to spreading confirmed the presence of disaggregated viable cells. The treated and untreated samples were also analysed by CLSM using Live/Dead staining BacLight viability kits according to Di Fermo et al.^38^. For the green and red cellular microbial amount, ten fields were analysed by three blended microbiologists. SEM observation was performed to evaluate the *C. albicans* S5 morphology in the biofilm with and without (Control) the CMFs treatments, as indicated in the SEM analysis section.

### Fluidity changes ***C. albicans*** S5 membrane

The CMFs effect on *C. albicans* S5 membrane fluidity was determined by assessing the Laurdan generalized polarization (GPexc) as previously described^26^. Briefly, the broth culture was standardized at an OD_600_ of 0.4 and divided in three aliquotes of 1.5 ml: blank, control an treated sample with CMFs. For the fluidity test, STRESS program was used. This program was chosen because it was the best performing one in the anti-biofilm assay. The treated sample was exposed to CMFs for 22 min and the blank and control groups did not receive any treatment. After, the brothcultures were centrifuged (9000 rpm, 8 min), washed twice with 15 mM Tris–HCl buffer (pH 7.4), the blank was resuspended with Tris–HCl buffer (pH 7.4) and the other two samples were resuspended in 10 µM of Laurdan, incubated in the dark at 37 °C with shaking (500 rpm) for 1.5 h and then analysed by Varian Cary Eclipse fluorescence spectrofluorometer (Agilent Technologies, Santa Clara, California, USA) to evaluate the Laurdan emission. The excitation GPexc was calculated using the following equation: GPexc = (I440 − I490)/(I440 + I490), where I440 and I490 are fluorescence intensities at 440 and 490 nm, respectively. Higher Laurdan GPexc values in respect to the control correspond to lower membrane fluidity.

### Permeability ***C. albicans*** S5 membrane

The CMFs capability to modify the *C. albicans* S5 membrane permeability was performed with propidium iodide (PI) because it is able to intercalate with bases of deoxyribonucleic acid (DNA) giving fluorescence when the membrane has been permeabilized. This DNA-bound PI fluoresces with excitation and emission at 544 nm and 620 nm, respectively. Briefly, the broth culture was standardized at an OD_600_ of 0.4 and divided in two aliquotes of 1.5 ml: control a treated sample with CMFs. For the permeability test, the STRESS program was used. This program was selected because it was the best performing one in the anti-biofilm assay. The treated sample was exposed to CMFs for 22 min and the blank and control groups did not receive the treatment. After the treatment, the cells were washed in PBS and incubated with PI (1.3 μg/ml) at 37 °C for 20 min in dark^40^. The PI fluorescence was measured at excitation and emission of 544 nm and 620 nm respectively, through a fluorescence spectrophotometer (Agilent Technologies, Santa Clara, California, USA). The increase in fluorescence intensity indicates the ruptured of bacterial membrane.

### hGFs viability assay

To evaluate the effects of CMFs on viability, CellTiter96 assay (3-(4,5-dimethylthiazolyl-2)-2,5-diphenyltetrazolium bromide) (MTS, Promega, Madison, WI, USA) were used in accordance to manufacturer^41^. 1 × 10^4^ cells/well were seeded in 96-well plates and were subjected to CMFs according to experimental design. The absorbance was measured at 490 nm using a microplate reader (Synergy H1 Hybrid BioTek Instruments). Cell viability was evaluated as a percentage compared to unexposed cells (CTR).

### hGFs toluidine-blue staining

To determine how CMFs affected the density and shape of the cells, toluidine-blue staining was employed^42^. According to the experimental plan, 2 × 10^4^ cells/well were seeded in 24-well plates and were subjected to CMFs. Thereafter, cells were fixed using 70% cold ethanol and stained with 1% toluidine blue and 1% borax (Sigma Aldrich). Cells were observed by an stereomicroscope connected with a camera at 25× (Leica,Wild Heer-brugg, Wetzlar, Germany).

### hGFs wound healing assay

hGFs were seeded at a density of 35,000 cells/well and were cultured until the confluence. A scratch was made in each well using a 200 μl pipette tip. Medium was removed and cells were washed in PBS. Then, hGFs were exposed to CMFs in accordance with the experimental design. After 0, 24, 48 h following the scratch, photos were taken with a camera connected to an inverted optical microscope (Leica) at 4× of magnification. The wound aerea expressed in µm^2^ and the percentages of wound closure were measured using the software ImageJ 1.52 q (National Institute of Health, Bethesda, MD, USA)^43^.

### hGFs ROS levels

10^4^ cells/ well were seeded in 96-well plates and after 24 h were exposed to CMFs according to experimental design. ROS levels were quantified at 3,6 and 24 h after the treatment using Cellular Reactive Oxygen Species Detection Assay Kit (Abcam, Cat No. ab186027, Cambridge, UK), according to the manufacturer’s protocol^41^. The fluorescence at λ ex/em 520/605 nm was measured by a microplate spectrofluorometer (Synergy H1 Hybrid BioTek Instruments).

### hGFs picrosirius red staining and spectrophotometric analysis

hGFs were cultured in 24 well/plates at a density of 5 x 10^4^ cells/well. After the fixation with glutaraldehyde at 2.5% for 2 h, cells were incubated with the staining solution (Sigma Aldrich) at room temperature for 1 h. After the removal of the staining solution, the cells underwent three rounds of acetic acid washing (0.1% concentration). The images were then captured using a stereomicroscope (Leica) at 25×. Picro-Sirius red was eluted in 0.1 N sodium hydroxide, 200 l/well, and the plates were left at room temperature for 1 h for spectrophotometric examination^44^. The optical density (OD) was read at 540 nm microplate reader (Synergy H1 Hybrid BioTek Instruments).

### SEM analysis

Scanning electron microscope (SEM) was used to evaluate the morphology of *C. albicans* S5 and hGFs. *C. albicans* cultured in plates, while hGFs were seeded on titanium surfaces (Implacil, DeBortoli, São Paulo, Brazil). After the fixation with 2.5% glutaraldehyde for 1 h, samples were dehydrated using sequential concentrations of ethanol and sputtered with gold. They were observed at 300 and 3000× for hGFs and 1600× for *C. albicans* using SEM (EVO 50 XVP LaB6, Carl Zeiss, Cambridge, UK) at 10 kV^45^.

### Statistical analysis

Data has been collected from at least three independently conducted in triplace. The means ± standard deviation of the data were displayed. One-way analysis of variance (ANOVA) was used to evaluate differences between groups. Statistics were considered significant for P values ≤ 0.05.

## Data Availability

The data that support the fndings of this study are available from the corresponding author upon reasonable request.
